# Supplementary material for: Interdependent iron and phosphorus availability controls photosynthesis through retrograde signaling
Source: Nat Commun. 2021 Dec 10;12:7211. doi: 10.1038/s41467-021-27548-2 (PMC8664907; doi:10.1038/s41467-021-27548-2)
Supplement: Supplementary file 1 — Supplementary Information [file 41467_2021_27548_MOESM1_ESM.pdf]

Supplementary information for

Interdependent Iron and Phosphorus Availability Controls Photosynthesis Through Retrograde Signaling

Nam et al.

This file includes:

Supplementary Table 1

Supplementary Figs. 1 – 12

Supplementary Table 1. List of primers used in this study.

Gene_ID	5' Primer Sequence	3' Primer Sequence
At1g06980	AGGCGGAGAAAGGACACTC	GGCGATGACGGTGCTCTT
At1g13600	TGAACACGATTCCAGCCGA	TGATCAGACCATTGAGGTGGTAG
At1g32520	ATTGGCTCGAGGATTGTG	ATTTAGCCACCCTCAAATCAC
At1g50110	GCTGATGATTCTATGTACTCAAACG	ATGAACGATGTCGTATTGGACCTT
At1g64360	AAACCAACTGAAATGGGCCG	TTAGTAATTAGGGAAGTGTTTGCGG
At1g76800	GGTTACGTTTGGCTTGAC	TAGTAATCTTTGTTTGCATGAAG
At1g80340	CTGGTGGTCAACGTCGGT	AAGTGAGAGATGGGTAGAGAGGA
At2g30570	TTTGCCTCCAATGGGTAAGAAG	TCTGTTGACATCCTCTCATCAACC
At2g39270	GAATCCCAGAGTCATGGGCAA	ATGCATCAAATAACCATAGTGGGAC
At2g40230	ACTTGCCATCGAATCAGGTC	TTAACGGCGTCATTTTGTTC
At2g44940	TCGGTCAACGATGTGAGTAATTCTG	ACGCCGAAGTCGAAGCAC
At3g21055	CCCATCACCTCACAAACCA	CCTCGCTTGGGCTCTTCTTC
At3g46780	AGAAAGAAGCAGAAGCTGCAA	TCACCACTGCTTTCTTGGGT
At3g55230	CCAATCCGCTCGACTTCT	AAACGGTATTCCATTCACCT
At3g61470	ACACTGGCACTGGTCCTA	TACGGGGAACCTTAATTTG
At3g61470	TCTAAAATGGAACGTACAAGCCGAG	CTTCTGCCCATCCGATCAAAA
At3g16120	AGATATCTGCAAGGTAATAATACAG	AAAATAGAGAAGATAACTCGTAAGG
At4g01070	GAGTGAGGAACAAGATGAAG	TGAGACAAACGAAGCAAAG
At4g04955	TAAAGGAAGCAAAAGGCA	CTTCCCACAATTTTTCTCT
At4g36060	CTTCCAGTTCGTTTGATCAGC	ACGGCTTGATTTTGGAATCTTG
At5g01530	ACCTCAGTGATCCACTCCACA	TTGAAAATGTACGACGAGTAGG
At5g06510	TGGCTGTAAACAGGCAAATC	CACGTTACACACAATACATCATCTG
At5g08030	ACTCTCAAAGAGCTGAAAACACTTG	GCTGATTCATGAAAACCGGA
At5g39530	TCGAGTAAACTCTATCTTTGTTGCC	GAATTTGGAAAATTTGCGCC
At5g47550	GCGTCGGTGGTTGGAGTC	TGTTAAAAGCTTAAAGGAAGCGACC
At5g49630	CAGGCTCCTTAGTGAAAA	GTTTAAGACAAGCAAGTAGAGG
At5g51710	TGGCGAGATTCGCAGTGAT	TCAGGCGAATCGTTTTCGG
At5g52570	TGGCAGCAGGACTATCAACAA	ACGACGAAACAAACGGTGAGAA
At5g59780	GGTCGCCTTTAGTCTAGGAT	CTGACTTGTATTACACATCTTTTC
At5g63850	CGACGTCGTAGGGATTTT	TTACATTATTCACATTTCCCC
At1g80340	GTTAACCACGTCCGATCTC	AAATGAGTAGCTTTGGTAGC
At1g51400	GCTGCAGCTGTGTGCTCCT	TGATCCTTACAAACCACACTGAG
At5g01600	AGTTTGGCATTTCGACCAG	AACCATCTTGAACACAAAGC
At2g39770	AGATCAAATCAAACATCTTGAAGCC	GAGACAAAACAGCAAACGACCC
At4g26850	TGAAGGAACCATAGTTCATCAGCAA	GCACAAAATGCCCACAACC
At3g02870	ATCCGGTAAAGATTTGGA	GAATTGAGTTTGATCATATAAGG
At1g13600-GFP	ATGAACACGATTCCAGCC	ACACAACTCAAAATCTCTGCCGC
At1g13600-g	CTTGCAGTGTCGACCGAAT	TTAACACAACTCAAAATCTCTGCCGC
At4g00370-g	CTAGTCGAGAATTTTTTCTCCGG	CATCCAGCTCATCATTCATCTCTTTCATTTTC
AT3G21055	TCCCATCACCTCACAAACC	ACCTTAGCCAAAGAACAAACC
AT1G51400	TCTTCATTTCTCCCTACCGTC	GCTCTGTTCTTGCTTCTTGTTC
AT3G46780	GATTCAGAACGCTTCAGAGAC	ACTTTCCTCACCTCTTTCTCC

pAT3G21055	TCGAGGTAATGAAGAAGAAC	TGCTTGATAGTTGATTCTACTCGC
pAT1G51400	TTCTTGTATTGTTAATAAAATTAG	TGCAATTGGTAAGTTGGATTCCC
pAT3G46780	ATGAAATTAAAATTTTATAAC	TAAGAGGAGGTCGACGAGCTTAGACG
pAT5G01530	CTTTATTACCGTATGGAAATAG	CTCCGGCTAATTGGGTTTTGTGATATTC
pAT2G30570	ATTATCGGGGAAGCTTTCTTATC	TTCTCTATTTGCTTCCCCTAACGACTGAGAG
bZIP58	CTGCAGATGAACACGATTCCAGCCGA	GGATCCTTAACACAACTCAAAATCTCTGCCG

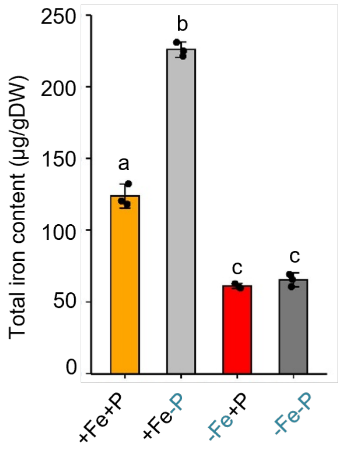

Supplementary Figure 1. Total iron content in response to Fe and/or P deficiency in *A. thaliana*. Mean iron content in shoots of fourteen-day-old *A. thaliana* plants grown on agar plates containing +Fe+P, +Fe-P, -Fe+P or -Fe-P. Data shown are from 3 experiments with 10 plants per experiment. Error bars represent 95% confidence intervals. DW: dry weight. Letters above bars represent statistically different means at $P < 0.05$ (one-way ANOVA with a Duncan post-hoc test). Source data are provided as a Source Data file.

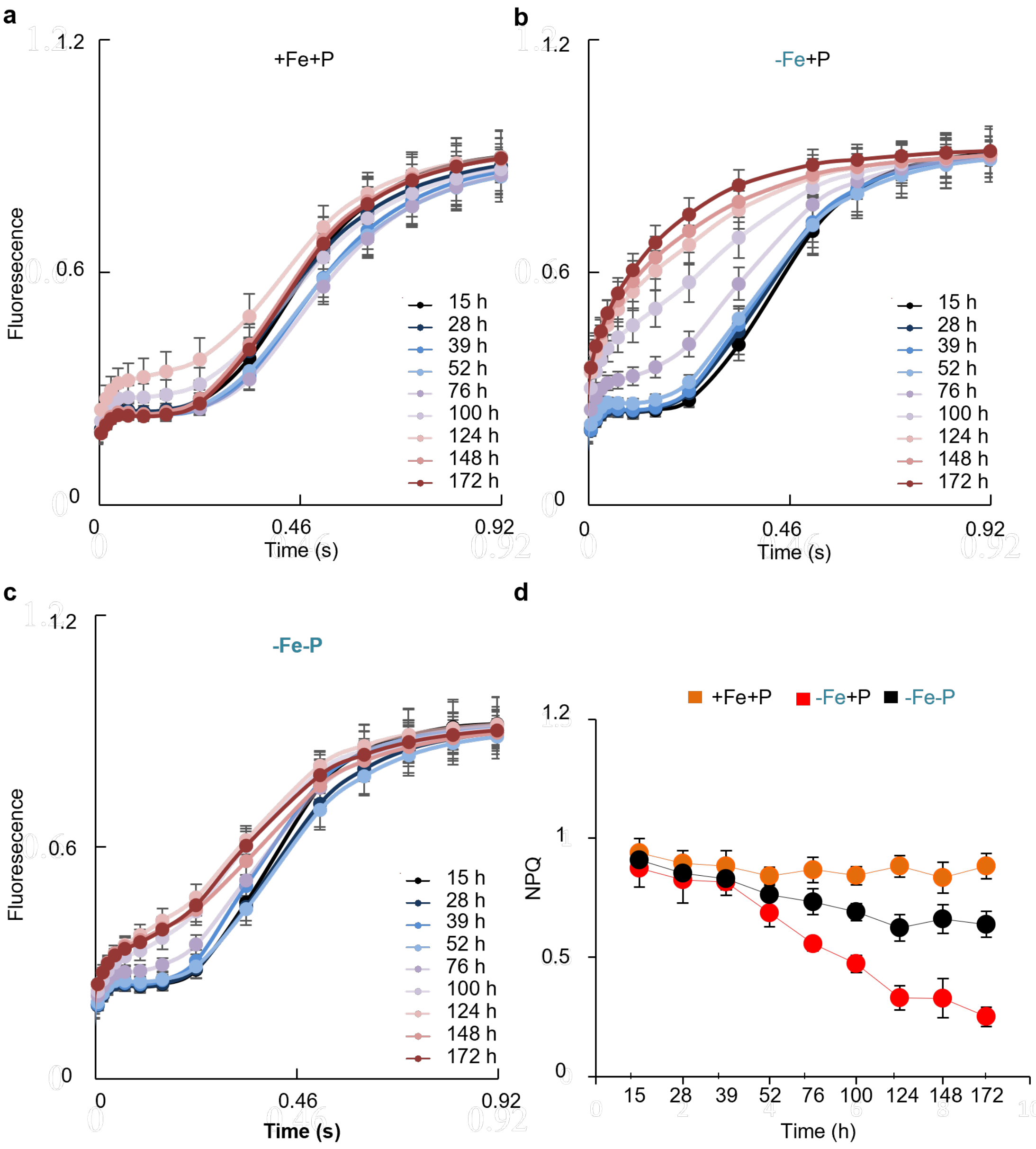

Supplementary Figure 2. Photosystem II activity in response to Fe and/or P deficiency in *A. thaliana*. (**A-D**) Kinetics of fluorescence (**A-C**) and nonphotochemical quenching (NPQ) (**D**) of seedlings grown for 7 days in the presence of Fe and P (+Fe+P) and transferred to three different media: +Fe+P (**A**), -Fe+P (**B**) or -Fe-P (**C**) for 15h, 28h, 39h, 52h, 76h, 100h, 124h, 148h, 172h. Data shown are the means from 3 experiments and 13 to 16 plants were measured per experiment. Error bars represent 95% confidence intervals. Source data are provided as a Source Data file.

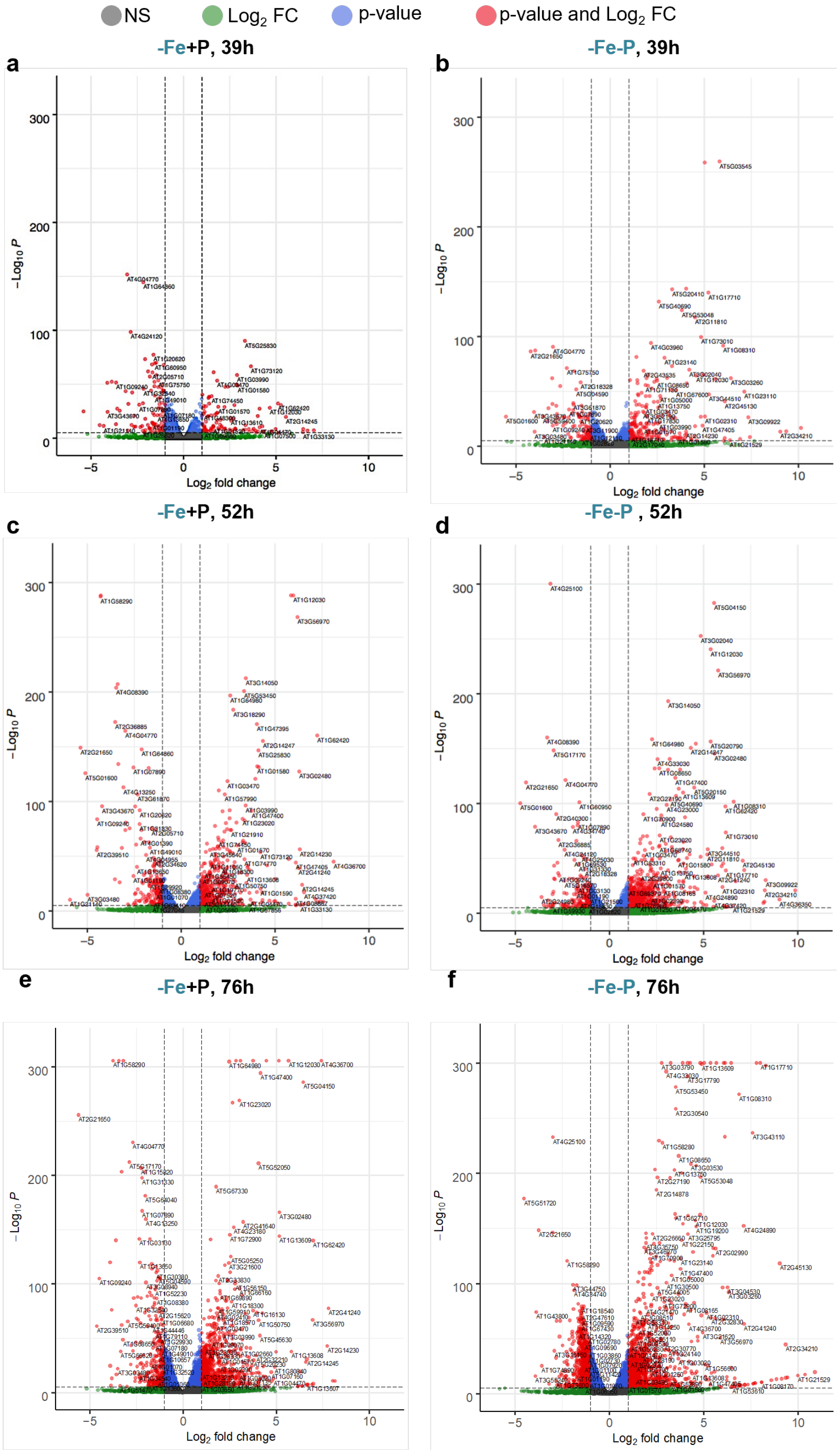

Supplementary Figure 3. Transcriptome kinetics of *A. thaliana* in response to Fe and/or P deficiency. (**A-F**) Volcano plots of individual transcript abundance in wild-type plants (Col-0) grown in -Fe+P (**A**, **C**, and **E**) or -Fe-P (**B**, **D**, and **F**) relative to +Fe+P. Shoot samples were collected from plants grown on +Fe+P for 7 days and transferred to each treatment for 39h (**A**, **B**), 52h (**C**, **D**), or 76h (**E**, **F**). x-axis: fold-changes; y-axis: adjusted p-values based on Benjamini-Hochberg correction; Both axes are in log scales. Red: $|\log_2 FoldChange|>1$ and $-\log_{10}P>6$; Blue: $|\log_2 FoldChange|<1$ and $-\log_{10}P>6$; Green: $|\log_2 FoldChange|>1$ and $-\log_{10}P<6$; Grey: $|\log_2 FoldChange|<1$ and $-\log_{10}P<6$. A default cut-off of $|\log_2 FoldChange|> 1$ and adjusted p-value < 10^{-6} was used.

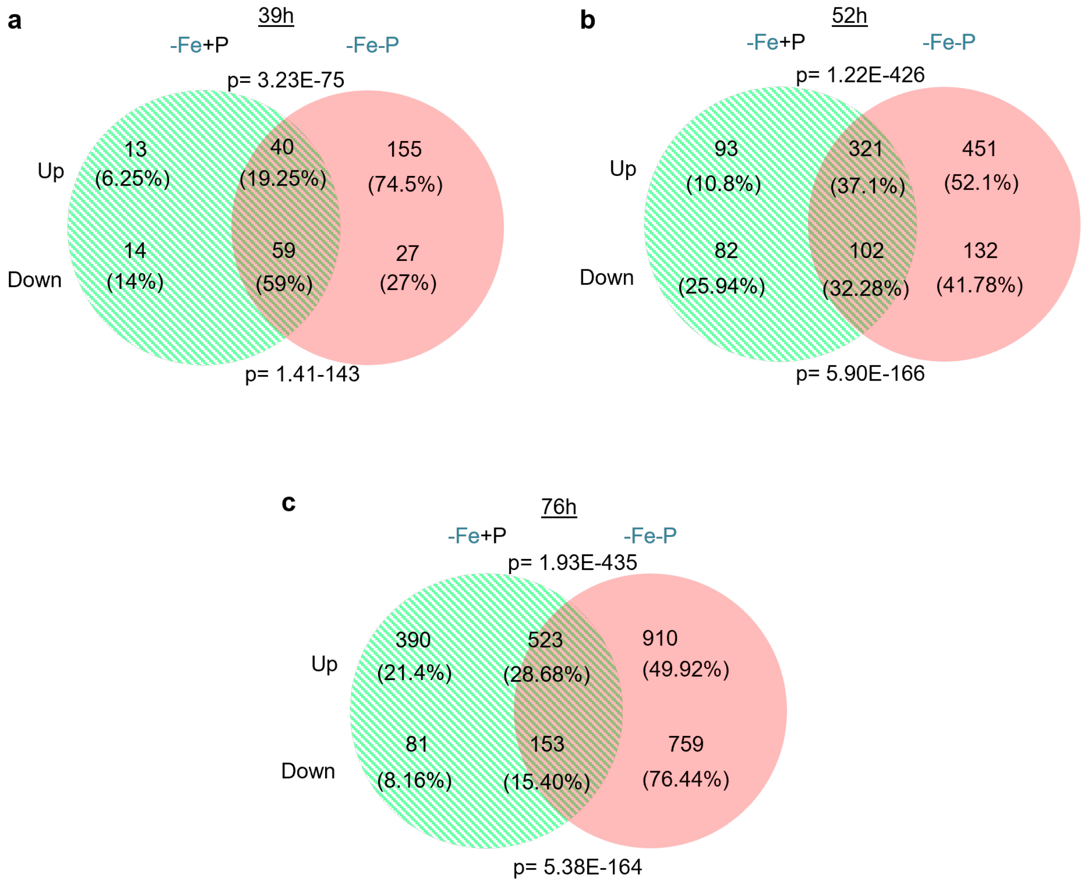

Supplementary Figure 4. Common and unique genes regulated by Fe and/or P deficiency. (**A-C**) Venn diagrams showing the number of differentially expressed genes in the shoots of *A. thaliana* wild type (Col-0) plants grown in +Fe+P for 7 days and transferred to -Fe+P or -Fe-P for 39h (**A**), 52h (**B**), or 76h (**C**) relative to those transferred to +Fe+P (fold change >2, $p < 0.05$). The Venn diagram was constructed using a web-based tool (http://bioinformatics.psb.ugent.be/webtools/Venn/). p = p-values from hypergeometric testing.

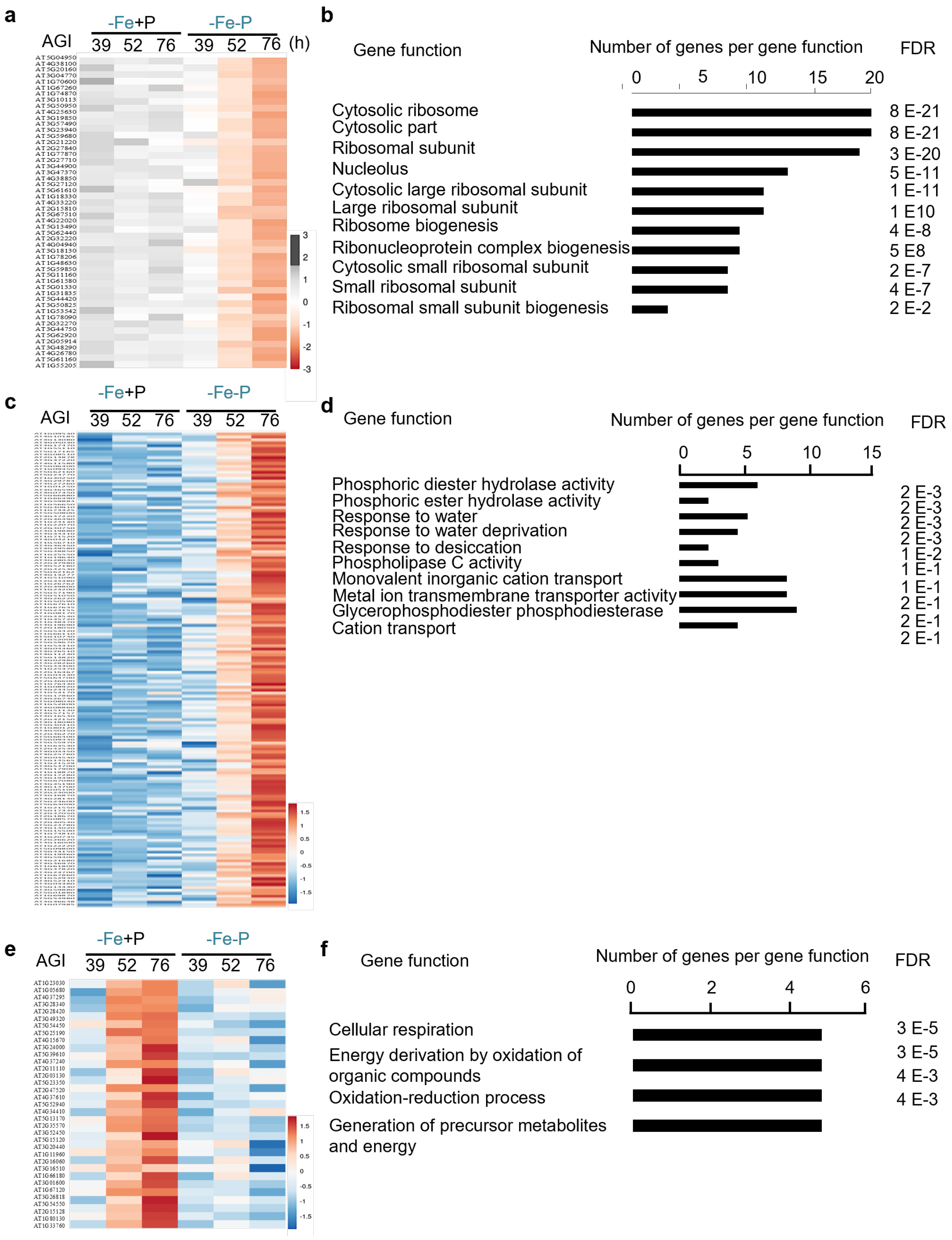

Supplementary Figure 5. Gene Ontology enrichment analysis of genes specifically regulated by Fe and/or P deficiency. (**A-F**) Heatmaps representing changes in the expression of genes that were specifically decreased (**A**) or increased (**C**) in abundance under -Fe-P but not -Fe+P relative to +Fe+P. A heatmap (**E**) representing the expression of genes specifically upregulated by -Fe+P but not by -Fe-P relative to +Fe+P. Gene Ontology enrichment for biological processes (GO-BP) in the genes that were specifically decreased by -Fe-P relative to +Fe+P (**B**), specifically increased by -Fe-P relative to +Fe+P (**D**), and specifically increased by -Fe+P relative to +Fe+P (**F**) using GENEMANIA[1]. Number of genes in each functional category and adjusted p-values for the enrichment are shown. FDR = false discovery rate.

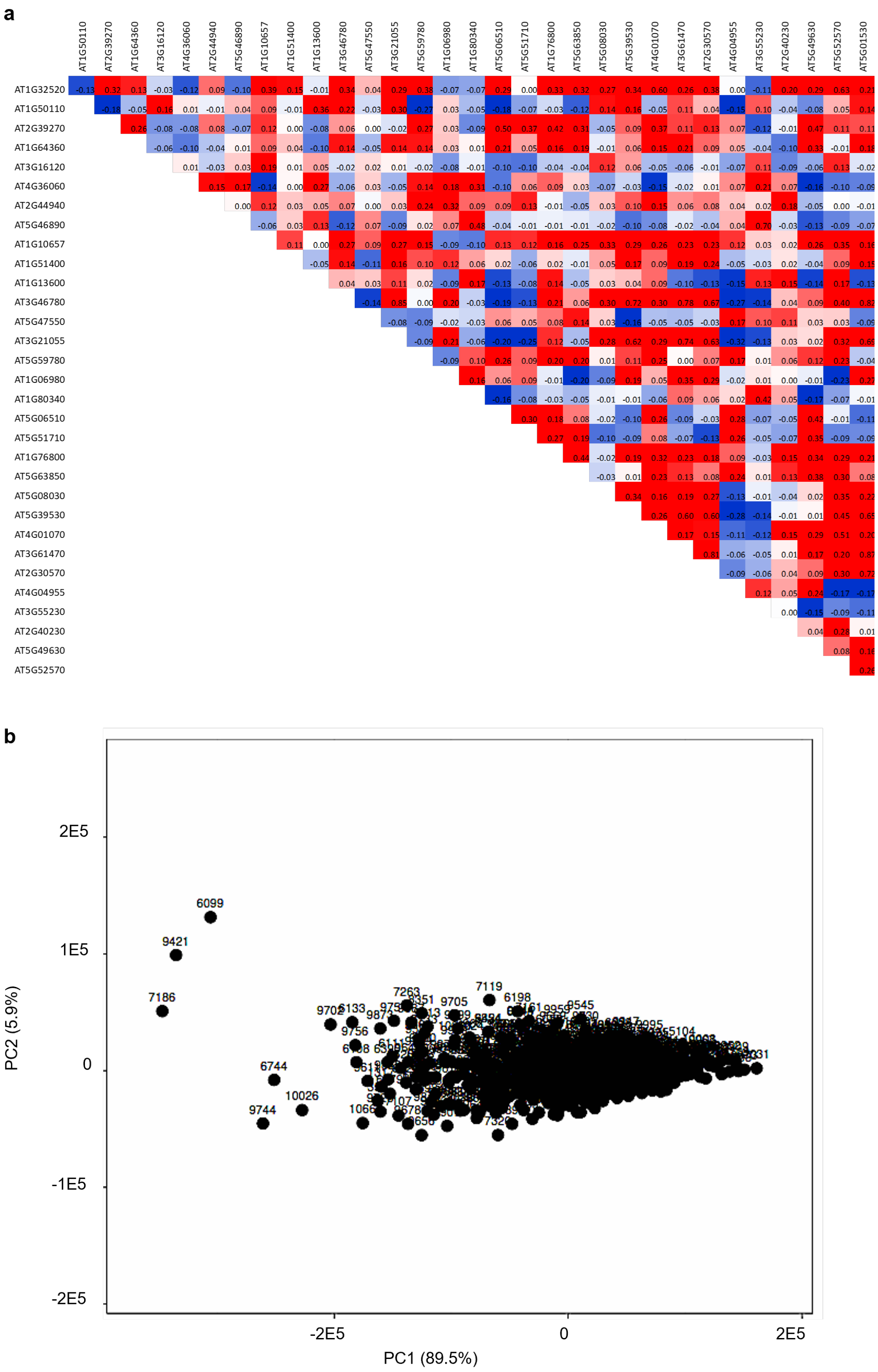

Supplementary Figure 6. Correlation in the expression of the 32 photosynthesis genes specifically downregulated by -Fe+P across 727 *A. thaliana* accessions. (**A**) A heatmap of pairwise correlations (Pearson's correlation coefficient) in the 32 genes across 727 Arabidopsis accessions grown under control conditions. The correlations were calculated using normalized read counts. (**B**) Principal Component (PC) Analysis performed using the expression of 32 genes in 727 Arabidopsis accessions. X- and Y-axes show PC 1 and PC 2 that explain 89.5% and 5.9 % of the total variance, respectively.

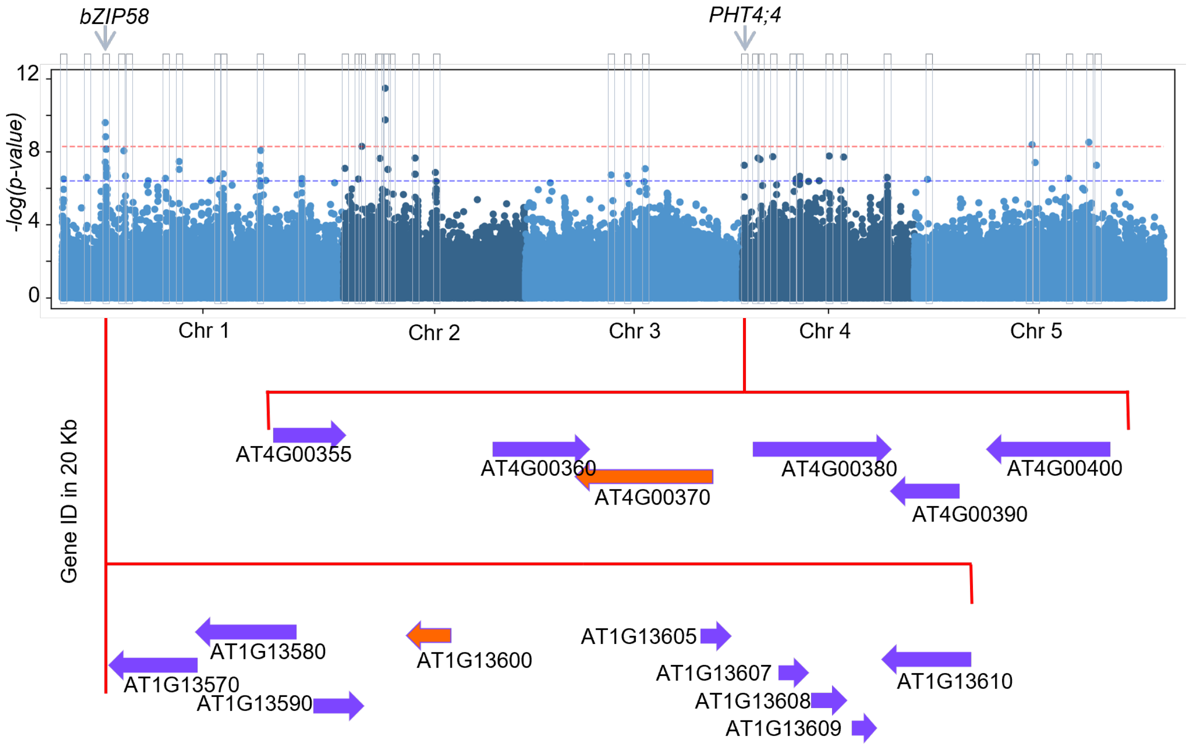

Supplementary Figure 7. A close-up view of chromosomes 1 and 4 around *bZIP58* and *PHT4;4*. A Manhattan plot for genome-wide association mapping using PC 1 of the expression profile of the 32 photosynthesis genes across 727 accessions. The five *A. thaliana* chromosomes are depicted by alternating light and dark blue colors. Blue and red horizontal dashed lines correspond to significant SNPs determined using an accelerated mixed model with adjustment for multiple tests using a FDR 5% threshold and Bonferroni α = 0.05, respectively. Light blue rectangles indicate the significant SNPs identified in this study. Below the Manhattan plot are gene models located within a 20-kb genomic region surrounding the two QTLs pursued in this study. The gene models of *bZIP58* (AT4G00370) and *PHT4;4* (AT1G13600) are indicated in red. Source data are provided as a Source Data file.

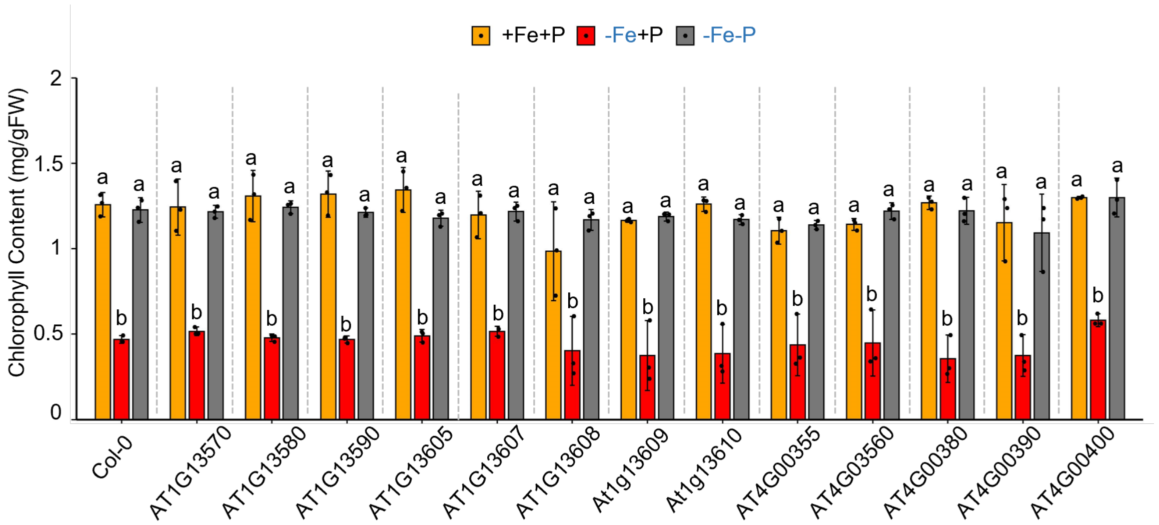

Supplementary Figure 8. Effects of Fe and/or P availability on chlorophyll content in *A. thaliana* Col-0 and mutants of candidate genes identified using GWAS. Mean chlorophyll content in Col-0 (CS60000), T-DNA insertion mutant lines in AT1G13570 (SALK_139877), AT1G13580 (SALK_150849), AT1G13590 (SALK_063177), AT1G13605 (SALK_087271), AT1G13607 (SALK_130208), AT1G13608 (SALK_023173), AT1G13609 (SAIL_1243_E04), AT1G13610 (SAIL_897_D11), AT4G00355 (N469134), AT4G00360 (SALK_128714), AT4G00380 (SAIL_842_E09), AT4G03585 (SALK_ 128714), AT4G00390 (SAIL_313_F07), and AT4G00400 (SAIL_633_E10) grown for 7 days in the presence of iron and phosphorus (+Fe+P) and transferred to +Fe+P, -Fe+P, or -Fe-P for an additional week. FW: fresh weight. Data shown from 3 experiments. Error bars represent 95% confidence intervals. Letters indicate significantly different values at $p < 0.05$ determined by one-way ANOVA and Tukey's honest significant difference (HSD) tests. Source data are provided as a Source Data file.

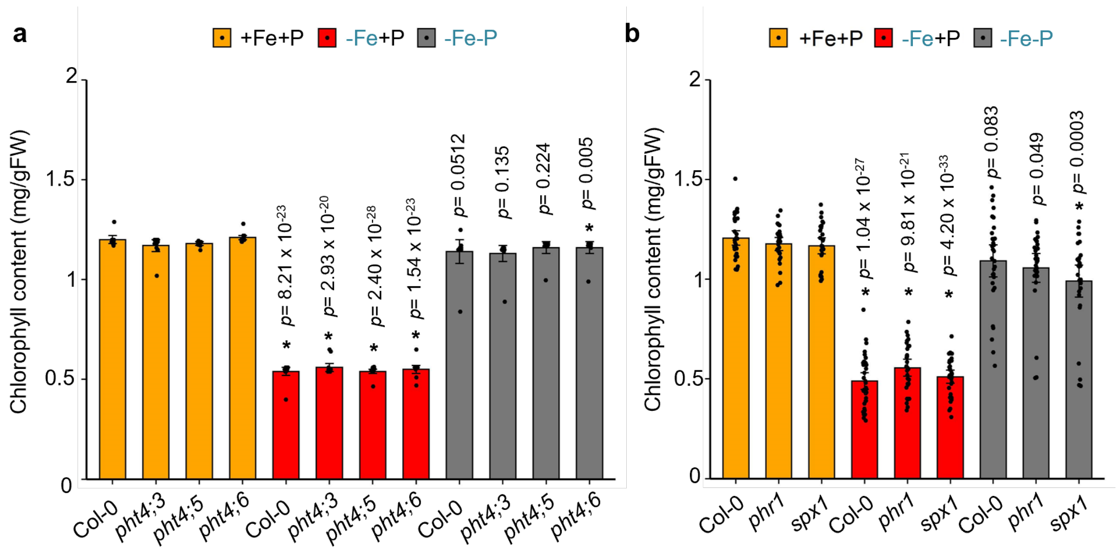

Supplementary Figure 9. Effects of Fe and/or P availability on chlorophyll content in *A. thaliana* Col-0 and mutants in phosphate-related genes. (**A**) Mean chlorophyll content in Col-0 (CS60000) and T-DNA insertion mutant lines *pht4;3* (SALK_053900), *pht4;5* (SALK_114708), and *pht4;6* (SAIL_809_B01). (**B**) Mean chlorophyll content in Col-0 (CS60000) and T-DNA insertion mutant lines *phr1* (SALK_067629) and *spx1* (SALK_039445). Plants were grown for 7 days in the presence of iron and phosphorus (+Fe+P) and transferred to +Fe+P, -Fe+P, or -Fe-P for an additional week. FW: fresh weight. Data shown from 3 experiments. Error bars represent 95% confidence intervals. Asterisks indicate that the relative chlorophyll content is statistically different from the Col-0 in control (+Fe+P) (p-value<0.01, two-tailed Student’s t-test). Source data are provided as a Source Data file.

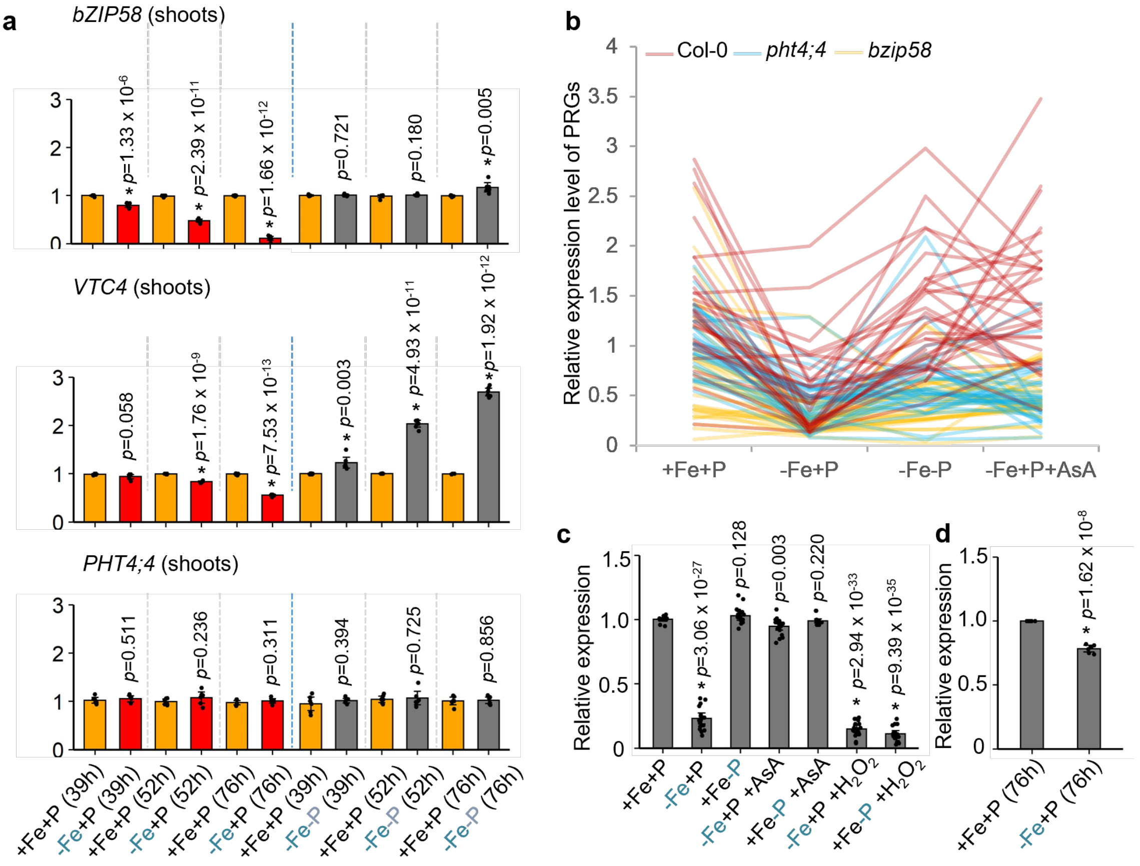

Supplementary Figure 10. Effects of Fe and/or P availability on the expression of *bZIP58*, *VTC4*, and *PHT4;4* in a time-dependent manner. (**a**) Relative mRNA abundance of *bZIP58*, *VTC4,* and *PHT4;4* in shoots of wild-type plants grown under +Fe+P for 7 days and transferred to +Fe+P, -Fe+P, -Fe-P for 39h, 52h and 76h. Data shown are the means of 6 biologically independent samples. Error bars represent 95% confidence intervals. (**b**) Relative mRNA abundance of individual PRGs in wild-type, *pht4;4*, and *bzip58* lines. (**c**) Relative mRNA abundance of *bZIP58* in shoots of wild-type plants grown in the presence of iron and phosphorus (+Fe+P) for 7 days and transferred to +Fe+P, -Fe+P, +Fe-P, -Fe+P+AsA, +Fe-P+AsA, -Fe+P+H_2O_2 and +Fe-P+H_2O_2 for 76h. Data shown are the means of 34 biologically independent samples. Error bars represent 95% confidence intervals. (**d**) Relative mRNA abundance of *bZIP58* in roots of wild-type plants grown under +Fe+P for 7 days then transferred to +Fe+P and –Fe+P for 76h. Data shown are the means of 6 biologically independent samples. Error bars represent 95% confidence intervals. Asterisks indicate significant differences in gene expression in the treatment compared to the +Fe+P control of the same time point (p-value<0.01, two tailed Student's t-test). Source data are provided as a Source Data file.

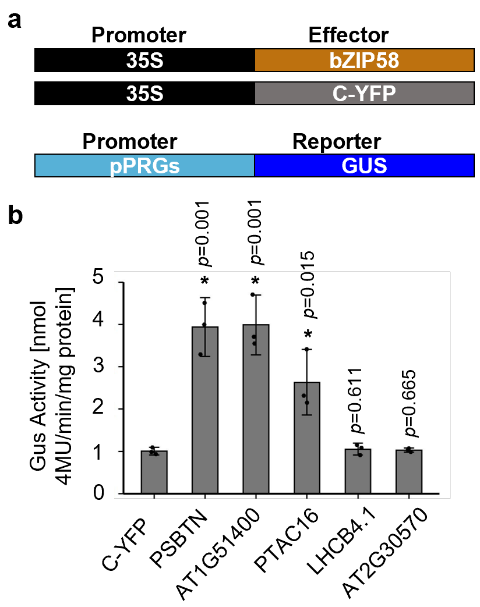

Supplementary Figure 11. ***In planta*** **transactivation assay**. (**A**) Schematic of effector (*35S::bZIP58* and *35S::C-YFP*) and reporter (*PRGp::GUS*) constructs used in the *in planta* transactivation assay. Promoters of the following PRGs were fused to the β-glucuronidase (GUS)-encoding reporter gene: *PHOTOSYSTEM II SUBUNIT T* (*PSBTN*), *PHOTOSYSTEM II 5 kD*, *PLASTID TRANSCRIPTIONALLY ACTIVE 16* (*PTAC16*), *LIGHT HARVESTING COMPLEX PHOTOSYSTEM II* (*LHCB4.1*) or *PHOTOSYSTEM II REACTION CENTER W* (*PSBW*). (**B**) Mean relative GUS activity for each promoter construct. Relative values were determined by the ratio of GUS activity when the PRG reporter construct was co-infiltrated with *35S::bZIP58* compared to when it was co-infiltrated with the *35S::C-YFP* control. Error bars represent 95% confidence intervals from three independent experiments. The asterisks indicate that the relative GUS activity is statistically different from the C-YFP control (p-value<0.01, two tailed Student's t-test). Source data are provided as a Source Data file.

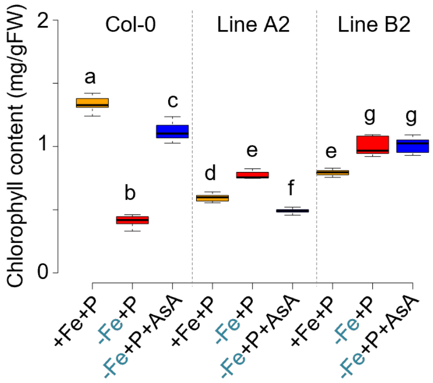

Supplementary Figure 12. Plastid ROS influences chlorophyll content. Total chlorophyll content in wild-type Col-0, a line expressing *JUB1p::bZIP58* in *bzip58* (Line A2), and a line expressing *JUB1p::bZIP58* in the *bzip58xpht4;4* double mutant (Line B2) grown for 1 week under +Fe+P and transferred to three different media conditions (+Fe+P, -Fe+P or -Fe+P+AsA) for 2 additional weeks. FW: fresh weight. Center lines show the medians of 10 biologically independent samples; box limits indicate the 25th and 75th percentiles; whiskers extend 1.5 times the interquartile range from the 25th and 75th percentiles. Letters indicate significantly different values at $p < 0.05$ determined by one-way ANOVA and Tukey's honest significant difference (HSD) tests.

References

1. Warde-Farley, D. *et al.* The GeneMANIA prediction server: biological network integration for gene prioritization and predicting gene function. *Nucleic Acids Res.* **38**, W214-W220 (2010)
