# Supplementary material for: Interdependent iron and phosphorus availability controls photosynthesis through retrograde signaling
Source: Nat Commun. 2021 Dec 10;12:7211. doi: 10.1038/s41467-021-27548-2 (PMC8664907; doi:10.1038/s41467-021-27548-2)
Supplement: Supplementary file 3 — Description of Additional Supplementary Files [file 41467_2021_27548_MOESM3_ESM.docx]

Description of Additional Supplementary Files

File name: Supplementary Data 1

Description: Differentially expressed genes responding to Fe deficiency and combined Fe and P deficiency.

File name: Supplementary Data 2

Description: Expression of 32 photosynthesis-related genes in bZIP58 and pht4;4 under Fe deficiency and combined Fe and P deficiency.
