# Supplementary material for: Interdependent iron and phosphorus availability controls photosynthesis through retrograde signaling
Source: Nat Commun. 2021 Dec 10;12:7211. doi: 10.1038/s41467-021-27548-2 (PMC8664907; doi:10.1038/s41467-021-27548-2)
Supplement: Supplementary file 6 — Reporting Summary [file 41467_2021_27548_MOESM6_ESM.pdf]

nature portfolio

Corresponding author(s): Seung Y. Rhee and Hatem Rouached

Last updated by author(s): Nov 5, 2021

Reporting Summary

Nature Portfolio wishes to improve the reproducibility of the work that we publish. This form provides structure for consistency and transparency in reporting. For further information on Nature Portfolio policies, see our Editorial Policies and the Editorial Policy Checklist.

Statistics

For all statistical analyses, confirm that the following items are present in the figure legend, table legend, main text, or Methods section.

n/a	Confirmed	
☐	☒	The exact sample size (n) for each experimental group/condition, given as a discrete number and unit of measurement
☐	☒	A statement on whether measurements were taken from distinct samples or whether the same sample was measured repeatedly
☐	☒	The statistical test(s) used AND whether they are one- or two-sided<br>*Only common tests should be described solely by name; describe more complex techniques in the Methods section.*
☒	☐	A description of all covariates tested
☒	☐	A description of any assumptions or corrections, such as tests of normality and adjustment for multiple comparisons
☐	☒	A full description of the statistical parameters including central tendency (e.g. means) or other basic estimates (e.g. regression coefficient) AND variation (e.g. standard deviation) or associated estimates of uncertainty (e.g. confidence intervals)
☒	☐	For null hypothesis testing, the test statistic (e.g. F, t, r) with confidence intervals, effect sizes, degrees of freedom and P value noted<br>*Give P values as exact values whenever suitable.*
☒	☐	For Bayesian analysis, information on the choice of priors and Markov chain Monte Carlo settings
☒	☐	For hierarchical and complex designs, identification of the appropriate level for tests and full reporting of outcomes
☒	☐	Estimates of effect sizes (e.g. Cohen's d, Pearson's r), indicating how they were calculated

Our web collection on statistics for biologists contains articles on many of the points above.

Software and code

Policy information about availability of computer code

Data collection	Chlorophyll content data were recorded using UV-VIS spectrophotometer (Beckman Coulter, DU 530), confocal laser-scanning microscopy was performed using Zeiss LSM510 , qRT-PCRs were performed in StepOnePlus RT-PCR system, iron quantification was performed using inductively coupled plasma-atomic emission spectrometry (ICP-OES), RNA sequencing was performed using the NextSeq 467 500 System at the Stanford Functional Genomics Facility (Stanford, CA).
Data analysis	QRT-PCR data were analysed using StepOnePlus sofftware v2.3, statistical analyses were performed using SigmaPlot 11.0 and R.4.0.3, RNA mapped read counts were used to perform normalization and a differential expression analysis on R using the DESeq. Volcano plots were generated using the EnhancedVolcano package (version 1.6.0)

For manuscripts utilizing custom algorithms or software that are central to the research but not yet described in published literature, software must be made available to editors and reviewers. We strongly encourage code deposition in a community repository (e.g. GitHub). See the Nature Portfolio guidelines for submitting code & software for further information.

Data

Policy information about availability of data

All manuscripts must include a data availability statement. This statement should provide the following information, where applicable:

- Accession codes, unique identifiers, or web links for publicly available datasets
- A description of any restrictions on data availability
- For clinical datasets or third party data, please ensure that the statement adheres to our policy

Data supporting the findings of this work are available within the paper and its Supplementary Information and Source Data files. The datasets and plant materials

generated and analyzed during the current study are available from the corresponding author H.R. upon request. Transcriptome data were deposited in NCBI's Gene Expression Omnibus (GEO) under a project number GSE163190 [https://www.ncbi.nlm.nih.gov/geo/query/acc.cgi?acc=GSE163190].

Field-specific reporting

Please select the one below that is the best fit for your research. If you are not sure, read the appropriate sections before making your selection.

☒ Life sciences ☐ Behavioural & social sciences ☐ Ecological, evolutionary & environmental sciences

For a reference copy of the document with all sections, see nature.com/documents/nr-reporting-summary-flat.pdf

Life sciences study design

All studies must disclose on these points even when the disclosure is negative.

Sample size	The chlorophyll contnet phenotypes of all genotypes were first investigated across various growth conditions based on a given number of plants (usually n> 10 plants). Tests of statistical power pointed to those lines or conditions that required more measurements to confirm a chlorophyll contnet phenotype to allow its detection at P < 0.05.
Data exclusions	No data were excluded from statistical analyses.
Replication	At least three independent experiments were performed for all genotypes in various growth conditions. The results were highly reproducible across experiments.
Randomization	To overcome heterogeneous growth conditions; such as light intensity in plant growth chambers, we grew the genotypes at random positions in the chamber and regularly changed positions throughout the growth.
Blinding	The experiments were not blinded as the differences were unamibguous. Also most of the experiments were performed by a single author that makes the blinding technically challenging.

Reporting for specific materials, systems and methods

We require information from authors about some types of materials, experimental systems and methods used in many studies. Here, indicate whether each material, system or method listed is relevant to your study. If you are not sure if a list item applies to your research, read the appropriate section before selecting a response.

Materials & experimental systems

n/a	Involved in the study	
☒	☐	Antibodies
☒	☐	Eukaryotic cell lines
☒	☐	Palaeontology and archaeology
☒	☐	Animals and other organisms
☒	☐	Human research participants
☒	☐	Clinical data
☒	☐	Dual use research of concern

Methods

n/a	Involved in the study	
☒	☐	ChIP-seq
☒	☐	Flow cytometry
☒	☐	MRI-based neuroimaging
